# Usual nutrient intake and adherence to dietary reference values among 3–6-year-old Finnish preschoolers

**DOI:** 10.29219/fnr.v70.13624

**Published:** 2026-06-23

**Authors:** Henna Peltonen, Maijaliisa Erkkola, Henna Vepsäläinen, Satu Kinnunen, Essi Skaffari, Kaija Nissinen, Eva Roos, Riitta Freese, Reetta Lehto, Jaakko Nevalainen, Liisa Korkalo

**Affiliations:** 1 Department of Food and Nutrition, University of Helsinki, Helsinki, Finland; 2 Department of Health Promotion and Care, Seinäjoki University of Applied Sciences, Seinäjoki, Finland; 3 Folkhälsan Research Center, Helsinki, Finland; 4 Department of Food Studies, Nutrition and Dietetics, Uppsala University, Uppsala, Sweden; 5 Department of Public Health, University of Helsinki, Helsinki, Finland; 6 Faculty of Social Sciences (Health Sciences), Tampere University, Tampere, Finland

**Keywords:** dietary survey, micronutrient intake, diet quality, dietary inadequacy, children, NCI, day-to-day variation, within-person variation

## Abstract

**Background:**

Early childhood is an important period for adopting a healthy diet to support long-term health. In Finland, data on how usual nutrient intake meets requirements in young children are lacking.

**Objective:**

We evaluated usual nutrient intake from food sources and adherence to dietary reference values among Finnish preschoolers.

**Design:**

We used cross-sectional data from the DAGIS survey conducted in 2015–2016 among 3–6-year-old Finnish preschoolers (n = 808). Dietary intake was assessed using 3‑day food records, complemented with a 2‑day food record in a subsample. National Cancer Institute methods were applied to estimate the usual intake distributions of macronutrients, vitamins and minerals for each age group (3-, 4-, 5- and 6-year-olds) and to assess the proportions of children below and above the most recent dietary reference values.

**Results:**

Mean protein intake was 17 E% in all age groups, and the fibre density of the diets averaged 2.5–2.6 g/MJ. High proportions of children had intakes above the recommended intake levels for saturated fatty acids (80–86% depending on age), free sugars (42–47%) and sodium (96–99%), and below the recommended intake level for polyunsaturated fatty acids (43–48%). High proportions of children had intakes below the average requirement for vitamin D (24–33%), vitamin E (61–76%) and iron (36% in 3-year-olds). Intakes above the tolerable upper intake level were detected for zinc (58% in 3-year-olds; 6.1–17% in 4–6-year-olds) and iodine (26% in 3-year-olds; 6.4–11% in 4–6-year-olds).

**Conclusions:**

Developing strategies to reduce particularly saturated fatty acid and sodium intakes in Finnish preschoolers is essential for early prevention of chronic diseases. Our results warrant regular monitoring of usual nutrient intake in young Finnish children to support timely and evidence-based decision-making.

## Popular scientific summary

- We assessed usual nutrient intake from food sources and adherence to dietary reference values in 3–6-year-old Finnish preschoolers.
- Diets of Finnish preschoolers were too high in saturated fatty acids, sodium and free sugars and too low in polyunsaturated fatty acids.
- Developing strategies to reduce particularly saturated fatty acid and sodium intakes in Finnish preschoolers is essential for early prevention of chronic diseases.

Early childhood is a critical period of life for adopting a healthy diet. Nutrient intake should support not only rapid growth and development (1, 2), but also long-term health and disease prevention. Diet-related chronic diseases typically arise during adulthood, but their risk factors, such as dyslipidaemia, elevated blood pressure and obesity, are often already present in childhood (3–6). Therefore, from a public health perspective, it is crucial to monitor nutrient intake in young children and identify targets for improvement.

When investigating the prevalence of inadequate or excessive nutrient intake, obtaining accurate results is particularly important for such vulnerable groups as young children, whose rapid growth and development make them sensitive to suboptimal nutrient intake. Although food records are considered a golden standard for the most precise measurement of nutrient intake in dietary surveys, this method typically records intakes over a short time frame, usually from one to a few days. Given that an individual’s dietary intake inherently varies from day to day also in young children (7, 8), short-term nutrient intakes are subject to high within-person variation and can misrepresent an individual’s usual nutrient intake, that is, long-term average daily intake (9, 10). The observed distribution of nutrient intake would therefore represent both within- and between-person variability, which in turn would lead to overestimation of the true levels of inadequate or excessive intakes (9, 10). However, improved estimates of usual nutrient intakes can be obtained by correcting short-term intake data for within-person variation (10).

Over the past couple of decades, several surveys among young children of high-income countries have applied usual intake estimation methods and reported excess intakes of saturated fat and sodium (11–21). By contrast, the intakes of vitamin D, vitamin E, calcium and iron have often been inadequate (11–20, 22–24). In Finland, monitoring nutrient intake of young children has not been carried out consistently. The most recent findings from a survey conducted in 2003–2005 among children aged 1–6 years indicated that mean intakes of saturated fat exceeded recommendations, whereas those of polyunsaturated fat, iron and vitamins D and E were below recommended levels (25). There is currently a lack of information to evaluate whether these concerns in nutrient intake continue in Finnish children and to provide accurate estimates of how dietary intakes meet reference values.

In this study, our aim was to estimate usual nutrient intakes for a wide range of macronutrients, vitamins and minerals and evaluate adherence to dietary reference values among 3–6-year-old Finnish preschoolers. Since meeting the nutritional needs of children should, in principle, rely on the consumption of food sources rather than supplements, we sought to understand how this is achieved and focused on nutrient intake from foods and beverages alone.

## Materials and methods

### Study design and participants

The study used data from the Increased Health and Wellbeing in Preschools (DAGIS) research project. DAGIS aimed to investigate energy balance-related behaviours, stress regulation and their determinants among 3–6-year-old Finnish preschoolers. The study protocol and description of the survey process are described in detail elsewhere (26, 27). Briefly, a cross-sectional survey was conducted in eight municipalities in Southern and Western Finland in 2015–2016, and recruitment was conducted through early childhood education and care centres (hereafter referred to as preschools). The eligibility criteria for preschools were 1) to have at least one group consisting of 3–6-year-old children, 2) to provide early childhood education only in the daytime, 3) to be Finnish or Swedish speaking and 4) to charge income-dependent fees. We contacted 169 preschools, 86 (51%) of which consented to participate. In these preschools, all children in the 3–6-year-old groups (*n* = 3,592) and their families were invited to participate through an invitation letter. We excluded preschools with a too low participation rate (i.e. parental consent rate was less than 30% in each of the preschool groups for the 3–6-year-olds), which left a total of 892 consenting children from 66 preschools. Of these children, 864 (24% of those invited) provided data for at least part of the survey and formed the final survey sample.

A parent or legal guardian of each participating child provided written informed consent. We also asked each family if they could be re-contacted for additional data collection. The study protocol was approved by the University of Helsinki Ethical Review Board in the Humanities and Social and Behavioural Sciences on 24 February 2015 (Statement 6/2015).

### Food record data

Data on children’s food consumption were collected in two separate periods using food records. Firstly, 3-day food records were collected between September 2015 and April 2016. Parents kept a food record for their child on 2 weekdays and 1 weekend day outside preschool hours. The dates were assigned by the research staff, and to improve representativeness of the data, the 3 days were not always consecutive. The assigned dates were also negotiable if families considered them unsuitable. Early educators recorded each child’s food consumption during preschool hours on the same weekdays as the parents kept food records. Secondly, to account for possible seasonal variation in the children’s diet, 2-day food records were collected between June 2016 and September 2016 among the families who agreed to be contacted for additional data collection (*n* = 709). For this second round, the families were assigned a week from which the parents could select 2 days for recording, with at least 1 day being preferably a weekday, and parents took the records with instructions to preschool when needed. The time between the two periods of food record collections ranged from 4 to 11 months.

The food record form included an instruction page and one example page. Parents were instructed to record the amounts of all foods and beverages that the child consumed outside preschool hours. They were also asked to record all ingredients of composite dishes and exact product names for packed food products. The parents estimated portion sizes using household measures, weighing, package labelling or the validated Children’s Food Picture Book specifically designed for the DAGIS survey (28) and provided together with the food record form.

Early educators were given a separate food record form that included predefined pages for eating occasions (breakfast, lunch, afternoon snack and possible additional snacks), as well as predefined rows for different parts of a meal such as main courses, side dishes (potatoes, pasta and rice) and salad at lunch. In addition to the written instructions, early educators received verbal instructions from the research staff for recording the amounts of all food and beverages that the child consumed during preschool hours. They were also given the Children’s Food Picture Book for estimating portion sizes or they could estimate the amounts eaten in household measures.

The research staff reviewed the returned food records and made telephone calls if important details of recorded foods or beverages were missing. We also asked preschool food services if they were willing to provide their recipes for preschool meals to allow more precise dietary intake calculations. Moreover, we requested details on food products used in preschools, such as the type of milk and fat spreads, from kitchen personnel.

We received 3-day food records from 850 children (98% of survey sample) during the first round of data collection, followed by 2-day food records from 206 children (29% of those invited) during the second round. We excluded those children from the data for whom the home part of the 3-day food record was missing (*n* = 37). We also omitted single food record days that showed unrealistically long breaks between consecutive meals (> 8 h) and the days when a child was younger than 3 years or 7 years or older, as our target age range was 3–6 years. As a result, the analyses included 808 3–6-year-old children (94% of survey sample) who had valid food record data from 1 to 5 days (Table 1).

**Table 1:** Background characteristics of Finnish preschoolers participating in the DAGIS dietary survey (2015–2016)

Characteristics	All *n* = 808	3-year-olds *n* = 167	4-year-olds *n* = 294	5-year-olds *n* = 283	6-year-olds *n* = 64
**Sex, *n* (%)**					
Girls	386 (48)	82 (49)	140 (48)	132 (47)	32 (50)
Boys	422 (52)	85 (51)	154 (52)	151 (53)	32 (50)
**Weight status^a^, *n* (%)**					
Underweight	62 (7.7)	16 (9.6)	26 (8.8)	18 (6.4)	2 (3.1)
Normal weight	609 (75)	131 (78)	212 (72)	217 (77)	49 (77)
Overweight or obesity	89 (11)	8 (4.8)	34 (12)	34 (12)	13 (20)
Missing information	48 (5.9)	12 (7.2)	22 (7.5)	14 (4.9)	0 (0.0)
**Food records, number of days, *n* (%)**					
1	7 (0.87)	1 (0.60)	2 (0.68)	4 (1.4)	0 (0.0)
2	34 (4.2)	7 (4.2)	16 (5.4)	8 (2.8)	3 (4.7)
3	575 (71)	109 (65)	208 (71)	205 (72)	53 (83)
4	7 (0.87)	2 (1.2)	4 (1.4)	0 (0.0)	1 (1.6)
5	185 (23)	48 (29)	64 (22)	66 (23)	7 (11)
**Diet, *n* (%)**					
Omnivorous	791 (98)	162 (97)	289 (98)	277 (98)	63 (98)
Vegetarian^b^	5 (0.62)	1 (0.60)	2 (0.68)	2 (0.71)	0 (0.0)
Missing information	12 (1.5)	4 (2.4)	3 (1.0)	4 (1.4)	1 (1.6)
**Highest educational level in the family, *n* (%)**					
Secondary school or lower	174 (22)	37 (22)	56 (19)	69 (24)	12 (19)
Bachelor’s degree or equivalent	337 (42)	72 (43)	128 (44)	113 (40)	24 (38)
Master’s degree or higher	293 (36)	57 (34)	107 (36)	101 (36)	28 (44)
Missing information	4 (0.50)	1 (0.60)	3 (1.0)	0 (0.0)	0 (0.0)

Data are presented for the total sample and according to subgroups based on children’s ages on the first food recording day.

a Children were classified according to the age- and sex-specific extended International Obesity Task Force (IOTF) body mass index cut-offs for weight status (42).

b Pesco-lacto-ovo-vegetarian or lacto-ovo-vegetarian diet.

### Computation of nutrient intake

To compute nutrient intake of the children, food record data (excluding supplements) were entered to AivoDiet dietary calculation software (version 2.2.0.0, Aivo Oy, Turku, Finland). The AivoDiet used the Finnish National Food Composition Database (Fineli), Release 16 (2013), which is maintained and continuously updated by the Finnish Institute for Health and Welfare. The database includes recipes for typical Finnish mixed diets. For each recorded meal, the research assistant selected a suitable recipe from the database, adapted an existing one or created a new recipe according to parents’ reports. When the requested recipes for preschool meals were provided by preschool food services, they were entered into the database. Otherwise, we made estimations based on recipes used in other municipalities. The salt content of home dishes was based on recipes available in the database, and unless otherwise reported by the parents, main dishes, pasta, rice, potatoes and porridge were assumed to have been cooked with salt. Vitamin D values of foods fortified with vitamin D (milk and liquid dairy products, fat spreads and non-dairy milk substitutes) were checked against products available on the market at the time of the survey.

The database reported the total contribution of vitamin A in food as retinol activity equivalents (RAE; 1 µg RAE = 1 μg retinol or 12 μg beta-carotene or 24 μg of other carotenoids). For vitamin E, the database reported values as alpha-tocopherol (1 mg vitamin E = 1 mg alpha-tocopherol); for folate, as the sum of folates calculated from folic acid and several folate compounds (i.e. as total folate, which does not correspond to dietary folate equivalents); for niacin, as niacin equivalents (1 mg niacin equivalents = 1 mg niacin or 60 mg tryptophan); and for carbohydrates, as available carbohydrates (i.e. those digested and absorbed, excluding dietary fibre).

We computed salt equivalents by multiplying sodium intakes by 2.54. Since the database did not distinguish added and free sugars from total sugars, we developed food-group specific formulas to estimate their intakes (Supplementary Table 1). In our estimations, added sugars were defined as sugar, syrup, glucose syrup, malt extract and malt syrup used as such or added during food preparation or manufacturing. Free sugars were defined as all added sugars, sugars naturally present in honey, syrups, fruit juices and fruit juice concentrates, and all sugars naturally present in dairy-alternative drinks (such as oat, soya, rice and nut-based drinks). In addition, we considered sugars from fruit, berry and vegetable purees and powders, where the cellular structure has been broken down, as free sugars. These definitions were adapted from those of Public Health England (29) and the Swedish National Dietary Survey (30). Briefly, each recorded food item was assigned to a food group, and relevant food groups were given specific formulas to estimate the amount of added and free sugars in each recorded food item. The estimation approach depended on the food type. For foods with a minimal amount of naturally occurring sugars, total sugar values were assumed to represent added or free sugars. For composite foods containing dairy, fruits or vegetables as ingredients, we estimated added and free sugars by subtracting lactose and/or fructose from the total sugar content, given that data on total sugars and intrinsic sugars were available. For other composite foods, we multiplied the total sugar content by estimated proportion of added or free sugars. These proportions were derived based on our review of frequently used foods within the food group. To estimate proportions, we calculated the sugar content from the corresponding recipes from Fineli or using the information on added sugar content reported by the manufacturer.

### Comparison of nutrient intake with dietary reference values

Unless otherwise stated, we used dietary reference values for macronutrients, vitamins and minerals available from the Nordic Nutrition Recommendations 2023 (NNR2023) (31), which also form the scientific basis for the Finnish national nutrition recommendations (32). To consider the recommended intake level of essential fatty acids, we summed the intakes of linoleic acid (LA) and alpha-linolenic acid (ALA). To consider the recommended intake level of *n*–3 polyunsaturated fatty acids (PUFAs), we summed the intakes of ALA, eicosapentaenoic acid (EPA) and docosahexaenoic acid (DHA). To evaluate the combined intake of EPA and DHA, we used age-group specific adequate intake ranges available from the Food and Agriculture Organization of the United Nations (FAO) (33). Inadequate intakes of vitamins and minerals were estimated based on age-group specific average requirement (AR) values for vitamin A, vitamin D, vitamin C, thiamin, riboflavin, niacin equivalents, vitamin B_6_, folate, calcium, iron and zinc. The AR is defined as ‘the average daily nutrient intake level that is estimated to meet the requirements of half of the individuals in a particular life-stage group in the general population’ and is usually used for evaluating adequacy of nutrient intake of groups of individuals (31). For vitamin E, vitamin K, vitamin B_12_, potassium, phosphorus, magnesium and iodine, NNR2023 could not determine ARs but suggested provisional ARs (i.e. approximations of AR derived as 0.8 x adequate intake) (31), which we used in the current work.

Intakes of vitamins and minerals above the tolerable upper intake levels (ULs) were estimated based on age-group specific cut-off values established by the European Food Safety Authority (EFSA) and, if not available, those determined by the Institute of Medicine (IOM) or National Academies of Sciences, Engineering, and Medicine (NASEM; IOM was renamed as the National Academy of Medicine and then integrated into the NASEM in 2015; we refer to this authority as IOM/NASEM hereafter). The UL refers to ‘the maximum level of total chronic daily intake of a nutrient from all sources judged to be unlikely to pose a risk of adverse health effects to humans’ (34). For vitamin A, vitamin B_6_, vitamin D, vitamin E, iodine and zinc, ULs were available from EFSA (34–38). For vitamin C, calcium, phosphorus and iron, ULs were available from IOM/NASEM (39–41).

### Background characteristics and weight status

Child’s sex, adherence to vegetarian diets and parents’ educational level were inquired via questionnaires. The parents reported whether their child followed any of the following diets: a vegetarian diet containing fish, milk and/or egg or a vegan diet (no animal-based products). These answers were combined into one vegetarian diet group due very low overall prevalence in the data (vegetarian diet containing fish, milk and egg: 0.37%; vegetarian diet containing milk and egg: 0.25%; others: 0.0%), and children not following any vegetarian diet were classified as omnivorous. Parents reported their educational level using the following six answer options: comprehensive school, vocational school, secondary school, bachelor’s degree or equivalent, master’s degree and licentiate/doctoral degree. These categories were then recoded into the following three levels: secondary school or lower, bachelor’s degree or equivalent and master’s degree or higher; the highest degree in the household was considered in this work.

Children’s weight and height were measured at the preschool by trained research staff after removal of shoes and heavy clothing. Body weight was measured using CAS portable bench scales (CAS PB-100/200). Height was measured using stadiometers (SECA 217). Body mass index was computed for each child by applying kg/m^2^. To determine children’s weight status (underweight, normal weight or overweight or obese), the international age- and sex-specific body mass index cut-offs were used (42).

### Statistical methods

#### Description of study population

Children’s background characteristics are presented as frequencies and percentages for the entire survey sample (*n* = 808) and by subgroups defined based on children’s ages (3, 4, 5 or 6 years) on the first food recording day.

#### Usual intake distributions

To account for within-person variation across food recording days and estimate usual intake distributions, we used mixed effects models and a quantile estimation procedure developed by the National Cancer Institute (NCI) (10). The NCI method estimates the usual intake distribution by separating and removing within-person variability from the total variability. This adjustment reduces measurement error inherent in short-term food records, making the resulting distribution a more accurate reflection of differences between individuals. We described the distributions with means and 10^th^ and 90^th^ percentiles. We also used the above-described dietary reference values as cut-off points to estimate the percentage of children meeting, remaining below, or exceeding the target ranges of intakes. We used two approaches for modelling, which are detailed below. Usual intake modelling was carried out by SAS software 9.4.

For absolute nutrient intakes (units as g/day, mg/day, or µg/day), we applied the MIXTRAN and DISTRIB macros (43). In the MIXTRAN, the model type ‘amount’ was fit for each nutrient. We included the covariates sex, age (3, 4, 5 or 6 years) and weekend effect as factors in all models. Saturday and Sunday were considered as weekend days, and to account for a weekend effect, a weight of 2/7 was used for food records kept on weekend days. The DISTRIB macro was then used to generate the usual intake distribution for each nutrient and for estimating the means and percentiles for the usual intakes as well as the percentage of those children below or above the entered cut-off points. For energy intake (MJ/day), the same modelling approach and covariates were applied using the MIXTRAN and DISTRIB macros.

Nutrient intakes contributing to energy intake or expressed relative to energy intake were estimated as the ratio of usual nutrient intake and usual energy intake (43). The *NLMixed* macros were used to first fit univariate models separately for the nutrient intake and energy intake and then to fit a joint bivariate model for the two intake variables. We included the covariates sex, age (3, 4, 5 or 6 years) and weekend effect as factors in all models, and weights of 2/7 and 5/7 were used for food records kept on weekend days and weekdays, respectively. The macro *Distrib_Bivariate* was then used to generate the distributions of usual nutrient and energy intakes, followed by the macro *Percentiles_Survey*, which produced the means and percentiles for the ratio of usual intakes and estimated the percentages below, within or above the entered cut-off points. For usual macronutrient intakes, the unit was E% (percentage of usual energy intake from usual macronutrient intake), assuming that one gram of fat yields 37 kJ of energy and one gram of available carbohydrates and protein yields 17 kJ of energy. For dietary fibre, niacin equivalents and thiamin, the unit was g/MJ or mg/MJ.

The usual intake distributions and percentages meeting and not meeting the target intakes were estimated for the analytical sample and by age groups. The age groups for modelling were based on children’s current ages, that is, for each child, we computed their age in years on each food record day, and usual intake distributions for age groups were analysed according to the current ages (3, 4, 5 or 6 years). As a result, 134 children contributed data to two distinct age groups because of turning older during or in between the food record periods (40 children turned 4 years old, 53 children turned 5 years old and 41 children turned 6 years old). The analytical sample hence comprised 942 observations from 808 children. This approach was applied because part of the dietary reference values had been set for different age groups, and it was inevitable that the children became older during the collection of food records (there was a 4- to 11-month gap between the first and second period of food record collections in our survey).

## Results

### Participant characteristics

Most children were 4 or 5 years of age (71%), normal weight (75%) and came from families with the highest education level being a bachelor’s degree or higher (78%; Table 1). The prevalence of children following any vegetarian diet was very low (0.62%).

### Usual intake of energy, macronutrients and dietary fibre

Table 2 describes the estimated usual intake distributions of energy, macronutrients and dietary fibre for the analytical sample and by age groups. Table 3 presents compliance with the recommended intake levels.

**Table 2:** Usual intake distributions of energy, macronutrients, dietary fibre, sodium and salt in Finnish preschoolers (DAGIS survey 2015–2016; *n* = 808)

Energy and nutrients	Unit	All *n* = 942^a^			3-year-olds *n* = 167			4-year-olds *n* = 334			5-year-olds *n* = 336			6-year-olds *n* = 105		
		Mean	P10	P90	Mean	P10	P90	Mean	P10	P90	Mean	P10	P90	Mean	P10	P90
Energy	MJ	5.8	4.7	6.8	5.3	4.4	6.2	5.6	4.7	6.6	6.0	5.1	7.0	6.3	5.3	7.3
Protein	g	55.8	42.9	69.2	51.7	39.6	64.2	54.8	42.5	67.7	57.4	44.9	70.6	60.3	47.3	73.7
	E%	16.8	14.6	19.0	17.1	14.8	19.4	17.0	14.8	19.2	16.6	14.5	18.7	16.7	14.6	18.8
Carbohydrates^b^	g	165	134	199	152	124	180	160	131	190	174	143	205	179	148	211
	E%	48.7	44.1	53.4	48.9	44.1	53.7	48.3	43.7	53.0	49.0	44.4	53.6	48.5	44.1	53.0
Added sugars	g	30.6	18.2	44.4	27.8	16.3	40.5	29.0	17.3	42.0	32.0	19.5	45.9	35.4	22.1	50.0
	E%	7.8	4.8	11.1	7.6	4.6	10.9	7.7	4.7	10.9	7.9	4.9	11.1	8.3	5.3	11.6
Free sugars^c^	g	38.0	22.7	54.7	34.3	20.2	49.8	36.5	22.0	52.6	40.2	24.7	57.2	41.4	25.7	58.6
	E%	9.9	6.1	14.2	9.7	5.8	14.0	9.8	6.0	14.1	10.1	6.3	14.3	10.1	6.3	14.2
Dietary fibre	g	14.1	10.0	18.4	13.1	9.3	17.2	13.7	9.8	17.9	14.6	10.6	18.9	14.9	10.9	19.3
	g/MJ	2.6	2.0	3.2	2.6	2.0	3.3	2.6	2.0	3.2	2.6	2.0	3.2	2.5	2.0	3.1
Total fat	g	49.9	38.9	61.5	44.9	35.1	55.1	48.9	38.7	59.7	52.0	41.4	63.1	54.7	43.8	66.1
	E%	31.7	27.5	36.0	31.2	26.9	35.6	31.9	27.7	36.3	31.6	27.5	35.9	31.9	27.9	36.1
SAFAs	g	19.1	14.1	24.4	17.1	12.6	21.9	18.7	14.0	23.8	19.9	15.0	25.1	20.9	15.8	26.4
	E%	11.9	9.5	14.5	11.7	9.3	14.3	12.0	9.5	14.6	11.9	9.5	14.4	12.0	9.6	14.5
*Trans*-fatty acids	g	0.67	0.46	0.91	0.61	0.42	0.82	0.66	0.46	0.90	0.69	0.48	0.93	0.74	0.51	0.99
	E%	0.41	0.30	0.54	0.41	0.30	0.54	0.42	0.30	0.55	0.41	0.29	0.53	0.42	0.30	0.55
MUFAs	g	16.9	13.1	20.9	15.2	11.9	18.8	16.6	13.1	20.4	17.6	14.0	21.5	18.5	14.7	22.4
	E%	10.8	9.4	12.4	10.7	9.2	12.2	10.9	9.4	12.5	10.8	9.4	12.3	10.9	9.4	12.3
PUFAs	g	7.8	5.8	10.0	7.0	5.2	8.9	7.6	5.7	9.6	8.1	6.1	10.3	8.5	6.4	10.7
	E%	5.2	4.2	6.2	5.1	4.1	6.1	5.2	4.2	6.2	5.2	4.2	6.2	5.2	4.2	6.2
LA (18:2*n*–6)	g	5.2	3.8	6.7	4.6	3.4	5.9	5.0	3.7	6.4	5.4	4.0	6.9	5.7	4.3	7.3
	E%	3.4	2.7	4.2	3.4	2.7	4.1	3.4	2.7	4.2	3.4	2.7	4.2	3.5	2.8	4.3
ALA (18:3*n*–3)	g	1.5	1.0	2.0	1.3	0.94	1.8	1.4	1.0	1.9	1.5	1.1	2.0	1.6	1.2	2.1
	E%	1.0	0.77	1.3	1.0	0.76	1.3	1.0	0.78	1.3	1.0	0.78	1.3	1.0	0.77	1.3
LA + ALA	g	6.6	4.9	8.6	5.9	4.4	7.6	6.5	4.8	8.3	7.0	5.2	8.9	7.3	5.5	9.3
	E%	4.4	3.5	5.4	4.4	3.5	5.3	4.4	3.5	5.4	4.5	3.6	5.4	4.5	3.6	5.5
EPA (20:5*n*–3)	mg	75.3	37.0	122	71.3	34.6	116	75.0	37.0	122	76.5	37.8	124	78.3	38.8	127
	E%	0.07	0.03	0.11	0.06	0.03	0.10	0.07	0.03	0.11	0.07	0.03	0.11	0.07	0.03	0.11
DHA (22:6*n*–3)	mg	202	105	318	179	92.4	282	207	109	325	202	107	317	224	119	351
	E%	0.15	0.07	0.25	0.13	0.07	0.22	0.16	0.08	0.26	0.15	0.07	0.24	0.17	0.08	0.28
EPA + DHA	mg	270	123	454	238	107	400	274	125	461	272	125	457	299	138	502
	E%	0.22	0.10	0.36	0.18	0.09	0.30	0.23	0.11	0.38	0.21	0.10	0.35	0.24	0.12	0.41
*n*–3 PUFAs^d^	g	1.8	1.2	2.4	1.6	1.1	2.2	1.7	1.2	2.3	1.8	1.3	2.4	1.9	1.4	2.6
	E%	1.2	0.90	1.5	1.2	0.91	1.5	1.2	0.91	1.5	1.2	0.90	1.5	1.2	0.90	1.5
Sodium	g	2.0	1.5	2.4	1.8	1.4	2.2	1.9	1.5	2.4	2.0	1.6	2.5	2.1	1.7	2.6
	Salt equivalents^e^	g	5.0	3.9	6.2	4.6	3.6	5.6	4.9	3.9	6.0	5.2	4.1	6.3	5.5	4.4	6.7

Analytic sample and subgroups by age (some children contributed to two age groups due to ageing during data collection).

a A total of 134 children contributed data to two distinct age groups because of becoming older during the food recording days or periods (40 children turned 4 years old, 53 children turned 5 years old and 41 children turned 6 years old).

b Available (i.e. digested and absorbed) carbohydrates.

c Free sugars include added sugars; sugars naturally present in honey, syrups, fruit juices and fruit juice concentrates; sugars naturally present in dairy-alternative drinks (such as oat, soya, rice and nut-based drinks); and sugars naturally present in fruit, berry and vegetable purees and powders, where the cellular structure has been broken down.

d The sum of alpha-linolenic acid (18:3*n*–3), eicosapentaenoic acid (20:5*n*–3) and docosahexaenoic acid (22:6*n*–3) intake.

e Sodium intake (g) was multiplied by 2.54.

Abbreviations: ALA, alpha-linolenic acid; DHA, docosahexaenoic acid; EPA, eicosapentaenoic acid; LA, linoleic acid; MUFA, monounsaturated fatty acid; P10, 10th percentile; P90, 90th percentile; PUFA, polyunsaturated fatty acid; SAFA, saturated fatty acid.

**Table 3:** Compliance of Finnish preschoolers with the recommended daily intakes of macronutrients, dietary fibre and sodium (DAGIS survey 2015–2016; *n* = 808)

Nutrients	Intake level	All (%) *n* = 942^a^	3-year-olds (%) *n* = 167	4-year-olds (%) *n* = 334	5-year-olds (%) *n* = 336	6-year-olds (%) *n* = 105
Protein	10–20 E%	96.6	94.7	95.8	97.8	97.7
	< 10 E%	0.0	0.0	0.0	0.0	0.0
	> 20 E%	3.4	5.3	4.2	2.2	2.3
Carbohydrates	45–60 E%^b^	84.2	84.7	81.5	86.5	83.9
	< 45 E%	15.6	15.0	18.3	13.3	16.0
	> 60 E%	0.20	0.30	0.16	0.21	0.13
Added sugars	< 10 E%	82.2	83.7	83.5	81.8	77.2
	≥ 10 E%	17.8	16.3	16.5	18.2	22.8
Free sugars^c^	< 10 E%	54.9	57.7	56.3	53.0	53.0
	≥ 10 E%	45.1	42.3	43.7	47.0	47.0
Dietary fibre	≥ 2 g/MJ^d^	89.9	91.3	90.0	89.8	88.1
	< 2 g/MJ	10.1	8.7	10.0	10.2	11.9
Total fat	25–40 E%	97.3	96.5	97.3	97.6	97.8
	< 25 E%	1.7	2.8	1.5	1.6	1.2
	> 40 E%	0.96	0.80	1.2	0.79	0.96
SAFAs	< 10 E%	16.3	19.7	15.6	16.2	14.2
	≥ 10 E%	83.7	80.3	84.4	83.8	85.8
MUFAs	10–20 E%	75.6	70.9	77.7	75.3	76.8
	< 10 E%	24.4	29.1	22.3	24.7	23.2
	> 20 E%	0.0	0.0	0.0	0.0	0.0
PUFAs	5–10 E%	55.5	52.2	55.7	56.6	56.7
	< 5 E%	44.5	47.8	44.3	43.4	43.3
	> 10 E%	0.0	0.0	0.0	0.0	0.0
ALA	≥ 0.5 E%	99.9	99.9	99.9	99.9	99.9
	< 0.5 E%	0.06	0.09	0.06	0.05	0.04
LA + ALA	≥ 3 E%	98.7	98.2	98.7	98.8	99.0
	< 3 E%	1.3	1.8	1.3	1.2	0.99
EPA + DHA	3 years: ≥ 100 mg^e^ 4–6 years: ≥ 150 mg^e^	84.7	92.1	82.5	82.4	87.0
	3 years: < 100 mg 4–6 years: < 150 mg	15.3	7.9	17.5	17.6	13.0
*n*–3 PUFAs^f^	≥ 1 E%	78.7	79.4	79.3	77.8	78.7
	< 1 E%	21.3	20.6	20.7	22.2	21.3
Sodium	3 years: ≤ 1.1 g^g^ 4–6 years: ≤ 1.4 g^g^	2.4	0.6	4.2	2.0	1.0
	3 years: > 1.1 g 4–6 years: > 1.4 g	97.6	99.4	95.8	98.0	99.0

For each nutrient, the table presents the proportion (%) of children meeting the recommended intake level for the analytic sample and by age groups, followed by the proportions below or above the recommendation (some children contributed to two age groups due to ageing during data collection). Recommended intake levels are according to the Nordic Nutrition Recommendations 2023 (31), unless otherwise indicated.

a A total of 134 children contributed data to two distinct age groups because of becoming older during the food recording days or periods (40 children turned 4 years old, 53 children turned 5 years old and 41 children turned 6 years old).

b The recommended intake level refers to total carbohydrates including dietary fibre, whereas our data represent available (i.e. digested and absorbed) carbohydrates.

c Free sugars include added sugars; sugars naturally present in honey, syrups, fruit juices and fruit juice concentrates; sugars naturally present in dairy-alternative drinks (such as oat, soya, rice and nut-based drinks); and sugars naturally present in fruit, berry and vegetable purees and powders, where the cellular structure has been broken down.

d The lower bound of the recommended intake range of 2–3 g/MJ set by the Nordic Nutrition Recommendations 2023 (31).

e The lower bounds of the age-group specific adequate intake ranges (100–150 mg/day for 2–4-year-olds and 150–200 mg/day for 4–6-year-olds) set by the Food and Agriculture Organization of the United Nations (33).

f The sum of alpha-linolenic acid (18:3*n*–3), eicosapentaenoic acid (20:5*n*–3) and docosahexaenoic acid (22:6*n*–3).

g Chronic disease risk reduction intake level set by the Nordic Nutrition Recommendations 2023 (31).

Abbreviations: ALA, alpha-linolenic acid; DHA, docosahexaenoic acid; EPA, eicosapentaenoic acid; LA, linoleic acid; MUFA, monounsaturated fatty acid; PUFA, polyunsaturated fatty acid; SAFA, saturated fatty acid.

The mean energy intake ranged from 5.3 MJ/day in 3-year-olds to 6.3 MJ/day in 6-year-olds. The mean protein intake was estimated at 17 E% in all age groups. No child had protein intake below the recommended intake range of 10–20 E%, while 2.2–5.3% of the children, depending on age, had intakes above the upper end of the recommended range.

Mean carbohydrate intake was estimated at 48–49 E% across ages, and 13–18% of the children had intakes below the recommended intake range of 45–60 E%. The mean intakes of added and free sugars were estimated at 8 E% and 10 E%, respectively. The proportion of children exceeding the recommendation of < 10 E% ranged from 16% in 3-year-olds to 23% in 6-year-olds for added sugars and from 42% to 47% for free sugars. Fibre density of the diets averaged 2.5–2.6 g/MJ, and most children (88–91%) met the recommended level of ≥ 2 g/MJ.

For total fat, mean intakes were 31–32 E% between age groups, and the recommended intake range of 25–40 E% was met in almost all children (97–98%). The mean saturated fatty acid (SAFA) intake was estimated at 12 E% in all age groups. Most children (80–86%) did not meet the recommendation of < 10 E% for SAFA intake. The intakes of monounsaturated fatty acids (MUFAs) and PUFAs averaged 11 E% and 5.1–5.2 E%, respectively. Many children did not achieve the recommended minimum intake for MUFAs and PUFAs, with 22–29% having MUFA intake < 10 E% and 43–48% having PUFA intake < 5 E%. The intake of *trans*-fatty acids was negligible among the children.

Mean intake of essential fatty acids (LA + ALA) was 4.4–4.5 E% between age groups, and almost all children (98–99%) met the recommended intake of ≥ 3 E%. For ALA alone, the mean intake was 1.0 E% in all age groups, and nearly all children (99.9%) met the recommended intake of ≥ 0.5 E%. The total intake of *n*–3 PUFAs (ALA + EPA + DHA) averaged 1.2 E% in all age groups, and about one-fifth (21–22%) of the children did not reach the recommended intake of ≥ 1 E%. The mean intake of DHA ranged from 179 mg/day in 3-year-olds to 224 mg/day in 6-year-olds, and the intake of EPA + DHA ranged from 238 to 299 mg/day. Although the intake distributions were relatively wide, most children (82–92%) reached an adequate intake level of EPA + DHA.

### Usual intake of sodium and salt

Mean sodium intake ranged from 1.8 g/day in 3-year-olds to 2.1 g/day in 6-year-olds, corresponding to mean salt intakes of 4.6 to 5.5 g/day, respectively (Table 2). Almost all children (96–99%) exceeded the chronic disease risk reduction intake level for sodium (Table 3).

### Usual intake of vitamins and minerals

For most vitamins and minerals, the mean intakes tended to be higher in older age groups (Table 4). High proportions of children had intakes below the AR for vitamin D (24–33% between age groups), vitamin E (61–76% between age groups) and iron (36% among 3-year-olds) (Table 5). Among 4–6-year-olds, intakes below the AR were also observed for calcium (11–17%), folate (7.1–12%) and magnesium (5.2–13%). Intakes above the UL were detected for zinc (58% in 3-year-olds; 6.1–17% in 4–6-year-olds) and iodine (26% in 3-year-olds; 6.4–11% in 4–6-year-olds) (Table 6).

**Table 4:** Usual intake distributions of vitamins and minerals in Finnish preschoolers (DAGIS survey 2015–2016; *n* = 808)

Nutrients	All *n* = 942^a^			3-year-olds *n* = 167			4-year-olds *n* = 334			5-year-olds *n* = 336			6-year-olds *n* = 105		
	Mean	P10	P90	Mean	P10	P90	Mean	P10	P90	Mean	P10	P90	Mean	P10	P90
Vitamin A, μg RAE	545	354	766	498	323	697	544	355	762	562	367	785	574	376	802
Vitamin D, μg	9.3	5.8	13.0	8.9	5.5	12.5	9.1	5.7	12.8	9.6	6.1	13.4	9.3	5.9	13.0
Vitamin E, mg	6.3	4.6	8.1	5.7	4.2	7.4	6.1	4.5	7.9	6.5	4.8	8.4	6.7	5.0	8.6
Vitamin K, μg	57.8	40.4	77.1	50.6	35.6	67.3	57.4	40.7	76.0	60.6	43.0	80.0	61.3	43.6	81.0
Vitamin C, mg	69.2	41.3	101	62.4	36.9	91.4	67.5	40.6	98.4	73.1	44.4	106	73.5	44.9	106
Thiamin, mg	0.83	0.64	1.03	0.74	0.58	0.92	0.81	0.64	1.00	0.86	0.67	1.05	0.90	0.71	1.10
Thiamin, mg/MJ	0.15	0.13	0.17	0.15	0.13	0.17	0.15	0.13	0.17	0.15	0.13	0.17	0.15	0.13	0.17
Riboflavin, mg	1.6	1.1	2.2	1.5	1.0	2.1	1.6	1.1	2.2	1.7	1.2	2.2	1.7	1.2	2.3
Niacin eq., mg	19.5	15.0	24.2	17.9	13.8	22.1	19.2	14.9	23.7	20.1	15.7	24.7	20.8	16.4	25.6
Niacin eq., mg/MJ	3.4	3.0	3.9	3.5	3.0	3.9	3.5	3.0	3.9	3.4	3.0	3.8	3.4	3.0	3.8
Vitamin B_6_, mg	1.2	0.90	1.5	1.1	0.83	1.4	1.2	0.88	1.5	1.2	0.94	1.6	1.3	0.96	1.6
Folate^b^, μg	149	108	194	136	99	176	146	107	189	156	114	201	156	115	202
Vitamin B_12_, μg	4.3	2.8	6.0	4.1	2.7	5.7	4.3	2.8	5.9	4.4	2.9	6.1	4.4	2.9	6.1
Calcium, mg	956	639	1,288	897	587	1,220	941	630	1,270	983	665	1,315	1,008	688	1,344
Potassium, mg	2,544	1,933	3,182	2,351	1,771	2,949	2,505	1,916	3,126	2,627	2,024	3,255	2,712	2,099	3,357
Phosphorus, mg	1,133	844	1,437	1,057	779	1,344	1,114	834	1,411	1,168	881	1,468	1,206	914	1,514
Magnesium, mg	236	181	294	218	166	272	232	179	288	243	189	300	253	198	312
Iron, mg	7.2	5.3	9.2	6.6	4.9	8.3	7.0	5.3	8.9	7.4	5.6	9.4	7.8	5.9	9.9
Zinc, mg	7.9	6.1	9.8	7.3	5.6	9.1	7.7	6.0	9.6	8.1	6.3	10.0	8.6	6.7	10.5
Iodine, μg	186	134	242	175	124	228	183	132	238	191	139	247	197	144	254

Analytic sample and subgroups by age (some children contributed to two age groups due to ageing during data collection). Nutrient intakes were based on food sources.

a A total of 134 children contributed data to two distinct age groups because of becoming older during the food recording days or periods (40 children turned 4 years old, 53 children turned 5 years old and 41 children turned 6 years old).

b Folate is reported as total folate (i.e. the sum of folates calculated from folic acid and several folate compounds).

Abbreviations: eq., equivalents; P10, 10^th^ percentile; P90, 90^th^ percentile; RAE, retinol activity equivalents.

**Table 5:** Prevalence of Finnish preschoolers with usual vitamin and mineral intakes below the average requirement (DAGIS survey 2015–2016; *n* = 808)

Nutrients	AR	All *n* = 942^a^	3-year-olds *n* = 167	4-year-olds *n* = 334	5-year-olds *n* = 336	6-year-olds *n* = 105
		% below AR	% below AR	% below AR	% below AR	% below AR
Vitamin A	3 years: 240 μg RE/day^b^ 4–6 years: 270 μg RE/day^b^	1.1	1.1	1.4	1.0	0.82
Vitamin D	7.5 µg/day	28.2	33.0	30.0	24.2	27.1
Vitamin E	3 years: 6 mg/day^c^ 4–6 years: 7 mg/day^c^	68.1	62.1	75.6	65.8	60.8
Vitamin K	3 years: 10 µg/day^c^ 4–6 years: 15 µg/day^c^	0.0	0.0	0.0	0.0	0.0
Vitamin C	3 years: 20 mg/day 4–6 years: 30 mg/day	1.2	0.29	2.0	1.0	1.0
Thiamin	0.07 mg/MJ	0.0	0.0	0.0	0.0	0.0
Riboflavin	3 years: 0.5 mg/day 4–6 years: 0.6 mg/day	0.10	0.10	0.13	0.09	0.05
Niacin eq.	1.3 mg NE/MJ	0.0	0.0	0.0	0.0	0.0
Vitamin B_6_	3 years: 0.5 mg/day 4–6 years: 0.6 mg/day	0.07	0.03	0.12	0.05	0.04
Folate^d^	3 years: 90 µg/day 4–6 years: 110 µg/day	8.5	4.3	12.4	7.3	7.1
Vitamin B_12_	3 years: 1.2 µg/day^c^ 4–6 years: 1.4 µg/day^c^	0.01	0.01	0.02	0.01	0.01
Calcium	3 years: 400 mg/day 4–6 years: 700 mg/day	12.1	1.3	17.0	13.0	10.9
Potassium	3 years: 700 mg/day^c^ 4–6 years: 900 mg/day^c^	0.001	0.0	0.003	0.0	0.0
Phosphorus	3 years: 200 mg/day^c^ 4–6 years: 350 mg/day^c^	0.001	0.0	0.003	0.0	0.0
Magnesium	3 years: 136 mg/day^c^ 4–6 years: 184 mg/day^c^	8.1	1.5	12.6	7.9	5.2
Iron	3 years: 6 mg/day 4–6 years: 5 mg/day	9.9	36.4	6.1	3.1	2.0
Zinc	3 years: 3.8 mg/day 4–6 years: 4.8 mg/day	0.53	0.14	0.94	0.43	0.14
Iodine	80 µg/day^c^	0.13	0.28	0.13	0.09	0.05

Analytic sample and subgroups by age (some children contributed to two age groups due to ageing during data collection). ARs are according to the Nordic Nutrition Recommendations 2023 (31). Nutrient intakes were based on food sources.

a A total of 134 children contributed data to two distinct age groups because of becoming older during the food recording days or periods (40 children turned 4 years old, 53 children turned 5 years old and 41 children turned 6 years old).

b The AR for vitamin A was established for retinol equivalents (RE; conversion rate from beta-carotene to retinol is 6:1, and from other carotenoids 12:1), whereas our vitamin A intake data correspond to retinol activity equivalents (RAE; conversion rate from beta-carotene to retinol is 12:1, and from other carotenoids 24:1).

c Provisional AR derived as 0.8 x adequate intake (31).

d Folate is reported as total folate (i.e. the sum of folates calculated from folic acid and several folate compounds).

Abbreviations: AR, average requirement; eq., equivalents; NE, niacin equivalents; RE, retinol equivalents.

**Table 6:** Prevalence of Finnish preschoolers with usual vitamin and mineral intakes exceeding the tolerable upper intake level (DAGIS survey 2015–2016; *n* = 808)

Nutrients	UL	All *n* = 942^a^	3-year-olds *n* = 167	4-year-olds *n* = 334	5-year-olds *n* = 336	6-year-olds *n* = 105
		% above UL	% above UL	% above UL	% above UL	% above UL
Vitamin A	3 years: 800 μg RE/day^b,c^ 4–6 years: 1,100 μg RE/day^b,c^	1.3	4.0	0.61	0.74	0.97
Vitamin D	50 µg/day^c^	0.0	0.0	0.0	0.0	0.0
Vitamin E	3 years: 100 mg/day^c^ 4–6 years: 120 mg/day^c^	0.0	0.0	0.0	0.0	0.0
Vitamin C	3 years: 400 mg/day^d^ 4–6 years: 650 mg/day^d^	0.0	0.0	0.0	0.0	0.0
Vitamin B_6_	3 years: 3.2 mg/day^c^ 4–6 years: 4.5 mg/day^c^	0.0	0.0	0.0	0.0	0.0
Phosphorus	3,000 mg/day^d^	0.0	0.0	0.0	0.0	0.0
Calcium	2,500 mg/day^d^	0.0	0.0	0.0	0.0	0.0
Iron	40 mg/day^d^	0.0	0.0	0.0	0.0	0.0
Zinc	3 years: 7 mg/day^c^ 4–6 years: 10 mg/day^c^	17.8	57.5	6.1	10.2	16.6
Iodine	3 years: 200 µg/day^c^ 4–6 years: 250 µg/day^c^	11.3	25.9	6.4	9.1	11.2

Analytic sample and subgroups by age (some children contributed to two age groups due to ageing during data collection). Nutrient intakes were based on food sources.

a A total of 134 children contributed data to two distinct age groups because of becoming older during the food recording days or periods (40 children turned 4 years old, 53 children turned 5 years old and 41 children turned 6 years old).

b The UL for vitamin A was established for preformed vitamin A, whereas our vitamin A intake data correspond to retinol activity equivalents.

c Established by the European Food Safety Authority (34–38).

d Established by the Institute of Medicine/National Academies of Sciences, Engineering, and Medicine (39–41).

Abbreviations: RE, retinol equivalents; UL, tolerable upper intake level.

## Discussion

Our study provided a comprehensive view of nutrient intake and adherence to dietary reference values in Finnish preschoolers. Usual nutrient intakes were generally in line with target intakes for protein, total fat, carbohydrates, dietary fibre, essential fatty acids, EPA and DHA, and most vitamins and minerals. However, high proportions of children did not meet the recommended intake levels for sodium, SAFAs, PUFAs and free sugars. In addition, intakes below the AR were detected for vitamin D, vitamin E and iron.

No child had protein intake below the recommended minimum; if anything, high intakes were observed. Our results imply that the recommended protein intake level is readily met by Finnish preschoolers predominantly following an omnivorous diet, and there is no need to increase the intake from the current levels. Previous studies have suggested that higher protein intake during preschool years may increase the risk of subsequent overweight and obesity (44, 45). In contrast, carbohydrate intake was in the lower range of the recommended intake, and exceeding the recommendation for sugar intakes tended to show an age-related trend (ranging from 16% to 23% across ages for added sugars and from 42% to 47% for free sugars). Somewhat similarly in Ireland, mean free sugar intake ranged from 9 E% in 1-year-olds to 14 E% in 4-year-olds (17). The observed slight age trend may be a sign of children gradually adopting less favourable dietary habits as they grow older. High intakes of added and free sugars are associated with increased risk of overweight and obesity (46, 47), and the association with weight gain is suggested to be most consistent for free sugar sources (48). Therefore, high prevalence of excessive free sugar intake observed in our study raises concerns about long-term health outcomes.

Despite almost half of the children in our study having free sugar intakes exceeding the recommendation, it seemed that fibre intake was not compromised. The fibre density of the diets reached the recommended level in most children. Preschool meals consumed by our study population supported fibre intake, as they accounted for a significant proportion of whole-grain consumption and total fibre intake (49). Most of the fibre provided at preschool came from cereal products, mainly rye bread, crispbread, multigrain breads and porridge, while vegetables and fruits contributed smaller amounts (49).

While total fat intake was well within the recommend range, the dietary fatty acid composition was not in line with the recommendations for promoting long-term cardiovascular health. Roughly 80% of the children had SAFA intake above the recommended level. In contrast, approximately one-fourth of the children had MUFA intake below the recommended level, while nearly half demonstrated similarly low PUFA intake. Evidence supports increasing the intake of MUFAs and especially PUFAs as a replacement of SAFAs to reduce low-density lipoprotein cholesterol concentration (50) and lower the risk of coronary heart disease (51). Our results indicate that efforts to improve the quality of fatty acid intake in young Finnish children are warranted, considering that poor dietary fatty acid quality can initiate adverse effects already in childhood (4, 52).

Despite suboptimal dietary fatty acid composition between saturated and unsaturated fatty acids, nearly all children in our study met the recommendations for essential fatty acids, including ALA alone. Vegetable fats and oils, especially those made from rapeseed, are rich sources of ALA (32) and recommended for use on breads and for cooking in Finnish preschool settings (53). For example, the consumption of margarines, which contain rapeseed oil as an ingredient, was relatively high in our study population on weekdays (49) and likely supported ALA intake. The mean intakes of EPA + DHA (238–299 mg/day across age groups) were relatively high compared to preschool-aged children in Ireland (65–80 mg/day) (17), France (184 mg/day) (54) and the Netherlands (54 mg/day) (21). Fish, the primary source of these fatty acids, was consumed by approximately two-thirds of our study population during food record periods (55). This can explain the higher intake level in our study, but also reflect the proportion of children (8–18%) not reaching the adequate intake level for EPA + DHA. However, the observed prevalence should be viewed in light of ongoing uncertainty regarding optimal EPA and DHA intake since there are no agreed recommendations for their dietary intake in children (56, 57).

Approximately one in five children did not meet the recommended level for the total intake of *n*–3 PUFAs, although the 10^th^ percentiles of the usual intake distributions were close to the recommended level. Based on our results, we suggest that the NNR2023 recommendation for the total intake of *n*–3 PUFAs may not be fully applicable to children. Firstly, while the recommended intake of ALA (0.5 E%) is met, it is not realistic that children can obtain the remaining 0.5 E% from DHA and EPA alone. The combined intakes of EPA and DHA in the children of our study contributed to energy intake by 0.22 E% on average, even though the absolute intakes were relatively high. Secondly, EPA and DHA are not significant substrates for energy metabolism, as they exert more critical functions in neuronal and retinal development and are essential in numerous regulatory processes within the body (58, 59). Evaluating their intake on an absolute scale, rather than as a proportion of energy intake, is more appropriate.

Nearly all children had sodium intake greater than recommended for chronic disease risk reduction. Mean sodium intakes ranged from 1.8 g/day in 3-year-olds to 2.1 g/day in 6-year-olds. These intake levels are similar to previous data collected among Finnish children in 2003–2005, when the average sodium intake was reported as 1.8, 1.9 and 2.2 g/day for 3-, 4- and 6-year-olds, respectively (25). This implies that high salt intake had persisted in Finnish preschool-aged children over the years. Limiting salt intake in young children is crucial for preventing disease burden later in life (60). Childhood blood pressure tracks into adulthood (3), but even modest reductions in salt intake can lower blood pressure in children (60). We observed earlier that food services, which provide food for preschools, carry a great responsibility, as salt intake from preschool foods already accounted for the majority of children’s recommended daily maximum intake (49).

Mean intakes of vitamin D in the current study were 8.9 to 9.6 µg/day between age groups. Considerably lower dietary vitamin D intakes have been reported in children of other European countries, with mean intakes ranging from 1.4 to 3.6 µg/day (14, 17, 20, 21, 61, 62). We previously found that fortified dairy products and spreadable fats contributed most to vitamin D intake among the children (46 and 33%, respectively) (49). Taken together, our findings highlight the success of Finland’s general vitamin D fortification policy, which recommends routinely fortifying liquid dairy and fat spreads with vitamin D (63), and explains the higher dietary intake level in our child population. Still, inadequate dietary vitamin D intake was detected in nearly one-third of our children. This implies that the recommendation of year-round use of a vitamin D supplement for children in Finland is reasonable (32), and supplemental vitamin D is needed to support children achieving the target intake.

We observed a very high prevalence of vitamin E intakes below the AR among children (61–76% between age groups). Similar observations were made in Spanish, Greek and US preschool-aged children (11, 14, 18, 19, 22, 23). Lower vitamin E intakes in our study might occur in parallel with the observed low PUFA intakes, as both nutrients are abundant in the same food sources (i.e. plant oils, nuts and seeds). On the other hand, a survey among US children aged 4–6 years reported that although nearly 68% of their study population had usual vitamin E intake below the AR, fewer than 1% had serum alpha-tocopherol concentrations below 11.6 µmol/L (23), a level considered indicative of insufficiency (64). It seems that more data are needed to assess the relationships between dietary vitamin E intake, vitamin E status and critical measures for growth and development, before making strong conclusions on inadequate vitamin E intake in young children.

Iron intakes below the AR were relatively common in 3-year-olds (36%), unlike in older children (2–6%). Basal iron losses and thus total iron requirements are estimated to be higher for children aged 1–3 years than for those aged 4–6 years, which is why the NNR2023 guidelines set the AR for iron at 6 mg/day for younger children, compared with 5 mg/day for older children (31, 65). If iron intake does not meet physiological requirements, the body will utilise its iron stores (65). This raises concerns about whether especially children with intakes less than the AR have reduced iron stores. A survey among US children found that 11% of 1–3-year-old toddlers were iron-deficient based on serum ferritin concentrations, even though nearly all met the AR for iron intake (23). Monitoring iron status in young children should receive more attention, given that the absorption efficiency of iron is generally inefficient (66) and influenced by the type of iron (heme vs. non-heme) and other dietary compounds (67).

We observed intakes below the AR for calcium, folate and magnesium among 4–6-year-olds, with larger proportions than among 3-year-olds. This was likely due to the relatively large increase in the ARs for 4–6-year-olds. Still, we did not expect to see calcium intakes below the AR, as the consumption of milk and dairy products was relatively high in our study population, that is, roughly 500 g/day on average (68) and exceeding the current recommended consumption level for adults (31). When implementing the new NNR2023 guidelines recommending a predominantly plant-based diet with moderate dairy intake (31), it seems to be essential to also monitor calcium intake in children. On the other hand, a shift towards plant-based diets has the potential to improve folate intake. Our study population represented omnivorous children, and significantly higher dietary folate intakes have been observed in Finnish vegan and vegetarian preschoolers than in their omnivorous peers (69, 70). Usual magnesium intake level in our study population was higher than that reported in many European child populations (12, 17, 18, 20–22). We compared magnesium intakes with the provisional AR, which possibly overestimated the prevalence of dietary inadequacy in our sample.

Intakes exceeding the UL were detected for zinc and iodine. As the ULs for children were extrapolated from adult data (34), they resulted in values relatively close to the respective ARs, particularly for zinc. This likely explains why usual zinc intakes often exceed the UL in children (11, 14, 17, 18, 21, 24) and intakes above the UL do not appear to be associated with adverse health outcomes (71). Milk and dairy products are the main sources of iodine in Finnish preschoolers (49, 70), and their consumption was relatively high in our study population (68). Therefore, although the use of iodised salt is recommended in homes, food industry and mass caterings in Finland (72), efforts to reduce salt consumption are unlikely to compromise iodine nutrition in children. Nonetheless, it is important to periodically monitor urinary iodine.

The study’s primary strengths derive from its relatively large sample size and rigorous and validated methods. Accuracy of food records was improved by involving both parents and early educators and by using a validated picture book (28) for estimating children’s portion sizes. Collecting food records in two seasons enabled us to account for seasonal variation. Moreover, the use of a validated statistical method to estimate usual nutrient intake distributions reduced day-to-day variation and yielded more reliable estimates of adherence to target intakes. Finally, by developing specific formulas for added and free sugars, we were able to assess these intakes against recommendations, which would not have been possible using only the national Food Composition Database.

Our findings should be viewed in light of somewhat limited generalisability. Nutrient intake of Finnish preschoolers tends to be closer to the recommendations than that of children cared for at home (73), and our study sample represented mainly children of higher educated families who tend to have more favourable nutrient intakes than those of families with lower educated background (68). Therefore, our findings might be somewhat biased towards diets of better nutritional quality, which adds to the importance of regularly surveying nutrient intake in the Finnish child population in the future. In addition, lack of information on the saltiness level of home‑cooked foods and the type of salt used introduces some uncertainty in the estimated salt and iodine intakes; however, because milk and dairy products are the main iodine sources in Finnish preschoolers (49, 70), only part of the iodine intake is affected. When interpreting the current findings, it should also be noted that food selection has somewhat changed since our data collection, but its impact on children’s dietary patterns is likely to be gradual.

## Conclusions

The fibre density of the diets generally aligned with recommendations among 3–6-year-old Finnish preschoolers, and protein intakes were towards the upper end of the recommended range. However, the children’s diets were too high in SAFAs, sodium and free sugars and too low in PUFAs. Dietary intake of most vitamins and minerals was generally adequate, except for vitamins D and E, and for iron in 3-year-olds, for which inadequate intakes were common. Our findings indicate that developing strategies to reduce particularly SAFA and sodium intakes in Finnish preschoolers is essential for early prevention of chronic diseases. Our results also warrant regular monitoring of usual nutrient intake in young Finnish children to support timely and evidence-based decision-making.

## Supplementary Material


